# Use of a pressure-sensing walkway system for biometric assessment of gait characteristics in goats

**DOI:** 10.1371/journal.pone.0223771

**Published:** 2019-10-16

**Authors:** Rebecca E. Rifkin, Remigiusz M. Grzeskowiak, Pierre-Yves Mulon, H. Steve Adair, Alexandru S. Biris, Madhu Dhar, David E. Anderson

**Affiliations:** 1 Department of Large Animal Clinical Sciences, University of Tennessee College of Veterinary Medicine, Knoxville, Tennessee, United States of America; 2 Center for Integrative Nanotechnology Sciences, University of Arkansas at Little Rock, Little Rock, Arkansas, United States of America; Universita degli Studi di Napoli Federico II, ITALY

## Abstract

The purpose of this study was to quantitatively assess gait characteristics and weight-bearing forces during ambulation in goats free of lameness using a pressure-sensing walkway as a biometric tool for stride, gait, and force analysis. Forty-six non-lame adult goats ranging in age from 5 to 6 years, mixed-breeds, and with a mean body weight of 52 ± 7.1 kgs were used. Goats were trained to walk over a pressure-sensing walkway. Data for analysis was collected on 2 different days, 3 days apart. On each day, 2 to 5 walking passes, in the same direction, were captured for each goat. Data from 2 valid passes meeting the criteria for consistent walking gait on each day were averaged then used for analysis. Analysis was performed, including the day-effect, for stride, gait, and force characteristics. Of the 46 goats enrolled in the study, complete data sets were achieved in 33 (72%) goats. Gait biometrics were similar among the assessment days; therefore, all data was pooled for the purpose of characterizing data for individual limb and biometric parameter comparisons at the individual goat level. Statistical analysis revealed that no difference within the paired limbs, and that there were significant differences between the front limbs and hind limbs. Maximum force and maximum peak pressure were significantly greater for the front limbs as compared with the hind limbs (p < 0.001). Based on the results, gait and force characteristics can be consistently measured in goats using a pressure-sensing walkway during a consistent walking gait. Goats apply greater force to the forelimbs during the weight-bearing phase of stride as compared with the hind limbs. The use of objective assessment tools is expected to improve the ability of researchers and clinicians to monitor changes in weight bearing and gait and will contribute to improved animal welfare.

## Introduction

Goats are often chosen as a model for orthopedic research [1–4]. Advantages in the use of goats in research include ease of handling, ease of training, size, weight, and ambulatory characteristics relevant to translational research in humans [3, 4]. More detailed information concerning gait in goats is needed, especially when translation of data to implants and materials for use in people are needed, as goats have suitable metabolic and bone remodeling rates for translation to people [4]. Currently, subjective visual assessment of gait (Visual Lameness Score; VLS) is the standard of care in practice to assess lameness [5–12]. It is limited to use of a visual analog scoring, or numeric rating, with the gait of each animal being assigned a score [7]. There appears to be no validated standards for objective gait analysis in goats. Limitations associated with subjective gait analysis are numerous and include inter-observer variability, lack of a validated standard scoring system, and limitations associated with analysis of categorical data [8–12]. In other livestock species, such as cattle and horses, subjective visual assessment is shifting to objective assessment [13–16].

Objective lameness assessment currently relies on sensor technology, such as pressure sensing systems, force plates, accelerometers, and kinematic studies with 3D motion capture technology [16]. Devices used to monitor lameness are important tools that need to be precise and accurate [17]. Studies have shown that certain kinetic measurements may differ based on the device that is implemented, or even the method by which it is calibrated [18]. Weight bearing provides an important tool for assessing the functional use of the limbs. Most studies using objective gait assessment are aimed at gaining information related to lameness [14]. Lameness assessment research often is focused on the detection of severely lame animals, with less precision given to mild lameness [14]. Even minor dairy species, such as Mediterranean buffalo, are beginning to be looked at objectively for lameness, but problems arise with sensors that short, as continuous strides may be missed with a limited algorithm [19].

Difficulties associated with objective lameness technology become particularly relevant in orthopedic research, when new material or devices may rely on lameness assessment as an outcome parameter in terms of animal welfare [3, 4]. Many studies rely on subjective lameness scoring systems, only mention monitoring lameness post-operatively, and/or rarely report findings of lameness in study conclusions [20–22]. While several studies exist describing objective lameness outcomes in relation to orthopedic research, few are done from a basis of described normal variables available for that specific species [23, 24].

Advantages to pressure-sensing systems with walkways are that they are time efficient, can evaluate multiple sequential steps, and have the ability to evaluate the contralateral limbs within the same walking pass and in the same trial [18]. Although originally used in biped gait analysis, pressure-sensing mats have been used to study gait in multiple species, such as horses, cattle, turkeys, sheep, pigs, dogs, and cats [17, 18, 23–30]. However, few studies validate these measurements or describe normal biometric variables [17, 31]. In one study, normal intact Santa Ines sheep were evaluated using a pressure-sensitive walkway and a 3-camera kinematic system, which allowed for normal parameters to be described for varying age groups [31]. Literature describing pressure sensitive platforms for use in the biometric assessment of lameness has been described, but is lacking for goats initially free of lameness [32–34]. Sheep and goats are important models for orthopedic research and share many similarities [4]. Some aspects of gait analysis in sheep require more comprehensive assessment tools because of their flight zone and flocking behavior. These limitations can be addressed with training [4].

The purpose of this study was to quantitatively assess biometric variables of gait and associated forces during ambulation in goats free of lameness. We hypothesized that goats could be trained to walk across a pressure-sensing walkway to allow consistent recording of data for variables of stride, gait, and force. The objectives were to describe the characteristics associated with stride and weight-bearing force in goats free of lameness, thereby providing a baseline for future studies using a pressure-sensing mat investigating lameness. This information will not only benefit the veterinary community in providing a baseline for goats free of lameness, which may be applied to herd health lameness monitoring programs, but will provide a valuable set of gait parameters for goats as a reference in the design and conduction of orthopedic research where lameness is induced or is a concern.

## Materials and methods

### Goats

All study procedures were approved by the University of Tennessee Animal Care and Use Committee (protocol number 2383). Forty-six mixed breed goats between five and six years old and weighing 52 ± 7.1 kgs (range 40–69 kgs) were purchased from a licensed, commercial vendor. Goats were a mixed breed population, including Boer, Spanish, Nubian, Saanen, Oberhasli, and hybrid goats. Goats were judged to be free of lameness based on a visual lameness score of 0 (normal movement) out of 4 [2]. Hooves and feet were inspected, and hooves were trimmed to ensure all goats had normal and consistent conditioned feet. Goats were housed in groups of five to six in small pens (15 ft^2^ per animal) (National Research Council. *Guide for the care and use of laboratory animals*; National Academies Press, 2010). Flooring included a layer of wood shavings (2.5 to 5-cm thick) laid on top of rubber mats placed on top of a concrete floor in a conditioned housing facility for the duration of the study. They were fed a total mixed ration, provided access to hay as an environmental enrichment, and given access to automatic waterers to meet nutritional and metabolic requirements. Goats were weighed at study entry and exit to monitor nutrition and health.

### Data collection

**Fig 1:**
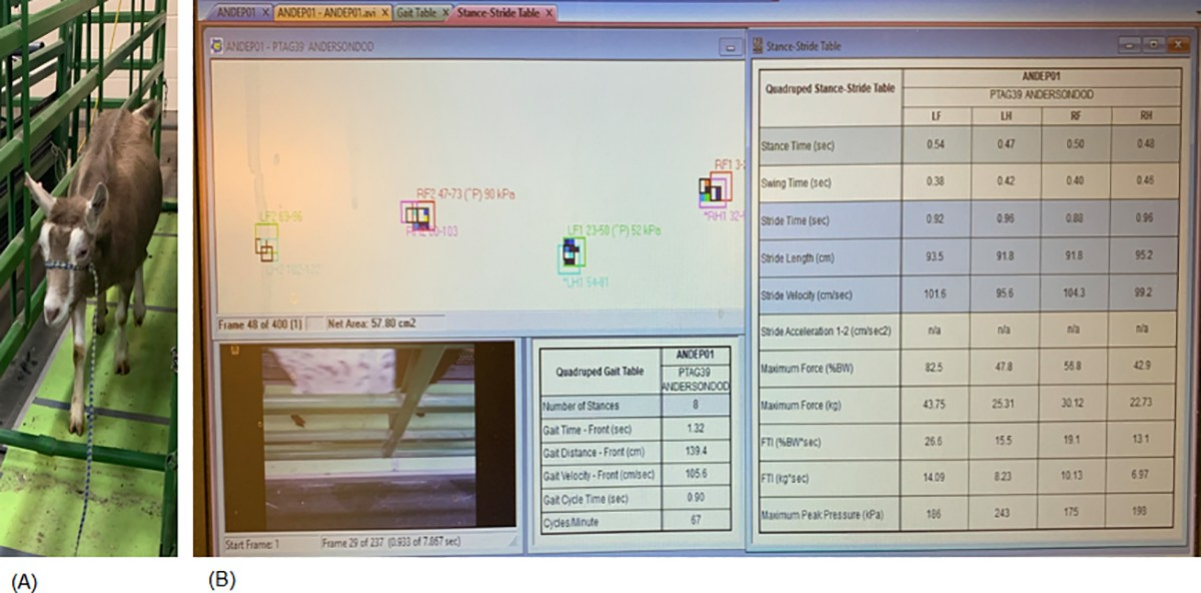
Examples of halter-lead training and sample gait analysis. (A) Pressure-sensing walkway placed in an alleyway system with soft mats and loose halter and lead for training. (B) Example of gait analysis with goat walking across pressure- sensing matrix placed in the alleyway system in the lower left-hand corner. The video recording with strike boxes is present in the upper left-hand corner, the stride stable is visible on the right, and the gait table is visible in the lower middle.

After confirming that all goats entering the study were free of lameness (VLS score of 0), gait parameters were objectively assessed by evaluating measurements obtained from an automated, real-time pressure sensing system (Walkway Pressure Mapping System, Tekscan Inc, South Boston, MA). The sensor matrix was 87.1 cm long by 36.9 cm wide and had a sensor density of 1.4 sensors/cm^2^. The mat was calibrated and equilibrated according to the manufacturer’s instructions. Triggering was enabled so that recordings would start at the first contact, or a raw sum force of 200 kPa, and end at total of 400 recorded frames at a rate of 15 frames per second. An alleyway was assembled in order to create a fixed walkway for the goats. The pressure mat was placed in the midpoint of the alleyway and covered with a soft, rubber overlay to create a consistent visual and tactile flooring with good footing for the testing area (Fig 1). The width of the alleyway was made such that goats could move freely in a straight line, would be discouraged from turning around, and that each goat’s footfalls would strike the sensitive area of the mat. Prior to initiation of the trial, goats were individually weighed using a digital scale. Goats were then fitted with a halter and led at a walking pace across the pressure-sensing mat, with an investigator sitting to the right of the mat adjacent to the constructed alleyway. The halter and lead were useful to encourage goats to pass through the walkway without stopping. During the study, no tension or pressure was applied to the halter to ensure that no changes in head movement or gait occurred.

Biometric assessment of gait was collected from the 2 best-fit recording in one direction, on each of 2 separate days (day 0 and 3). Each goat was walked across the mat up to 5 times (5 passes) until at least 2 valid walking passes were obtained. A pass was considered valid if the goat maintained a progressive walking gait, had a VLS of 0 during the pass, walked calmly through the alleyway without stopping or resisting walking forward, walked over the walkway without distraction, and if all four limbs had contact with the pressure sensing surface of the walkway. The data from the first two valid passes on each day were recorded and averaged for each goat, allowing for one set of data to be analyzed for each day.

Data was discarded during the data acquisition phase of the study if any goat hesitated, changed their gait or pace, or reacted to surroundings in a way that altered their gait. Acceptable behavior was limited to a subjectively assessed lameness score of 0, and walking in a forward manner without distraction from the investigator or hesitation from the halter and lead. Each pass was recorded with a digital video camera to record extremity strike and gait as the goat walked over the pressure sensor mat, and data from the mat sensor were transmitted to the system’s computer software (Microsoft LifeCam Cinema, Microsoft Corporation, Redmond, WA). The frame rate of the video camera was adjusted to 15 frames per second to match the pressure-sensing mat. Once data was transmitted to the software it was then exported to Microsoft Excel, backed up, and stored within an external hard-drive (Microsoft Excel for Windows 10, Seagate Portable 1TB External Hard Drive USB 3.0, USA). Due to significant range in the mixed breeds of the goats (Boer, Spanish, Nubian, Saanen, Oberhasli, and true mixes) and various limb lengths, it was necessary to average two valid passes to obtain consecutive footfalls, allowing for a complete data set for one valid run per day. Data sets were included in statistical analysis if they were complete for all variables.

#### Gait variables

Gait variables included the number of stance (or footfalls), gait time-front (sec), gait distance-front (cm), gait velocity-front (cm/sec), and cycles per minute. Stance and stride variables measured included stance time (sec), swing time (sec), stride time (sec), stride length (cm), stride velocity (cm/sec; Table 1). With goats that had long swing times, stride times, and/or stride lengths, multiple passes were required to obtain one valid run; therefore, the first two valid passes that allowed a complete data set were used in the analysis.

**Table 1:** Working definitions used for gait variables.

**Gait Variable**	**Definition**
Number of stances	The total number of stances (footfalls) taken by the animal.
Gait time-front (sec)	The time of first contact of the given front extremity stance to the time of first contact of the last given extremity stance as registered on the sensor.
Gait distance-front (cm), or length unit,	The gait distance measured along the line of progression, from posterior of the given front extremity stance to posterior of the last given extremity front stance.
Gait velocity-front (cm/sec)	The gait distance divided by the gait time.
Gait cycle time (sec)	Began with the first contact time of the given front extremity fall to be valid on the sensor. Time was measured to the first contact of the next instance of that given extremity striking the sensor.
Cycles per minute	The number of complete gait cycles per minute, or gait cycle time divided by sixty.
**Stand-Stride Variable**	**Definition**
Stance Time (sec)	The weight-bearing period, was defined as the time from first contact to last contact of a given extremity in seconds.
Swing Time (sec),	The non-weight bearing period, was defined as the elapsed time between the last contact of a preceding and the first contact of the next of two consecutive footfalls of a given extremity. When there were no consecutive footfalls, no data was recorded.
Stride Time (sec)	The elapsed time between the first contacts of two consecutive footfalls of a given extremity. If there were no consecutive footfalls, no data was recorded. If there were multiple strides within the same pass, times were averaged.
Stride Length (cm)	The distance measured parallel to the line of progression between the posterior heel points of two consecutive footfalls of a given extremity. When there were multiple strides, the lengths were averaged.
Stride Velocity (cm/sec)	The stride length divided by the stride time for the given extremity. When there were multiple strides, the velocities were averaged.

#### Force variables

Measurements of force variables included maximum force (Kg), maximum force normalized to body weight (%BW), impulse (Kg*sec), impulse normalized to body weight (%BW*sec), and maximum peak pressure (KPa). Maximum force (Kg) was the maximum force recorded during the stance phase of each extremity. Maximum force (%BW) was defined during the stance phase of the given extremity (normalized to the animal’s body weight). When there were multiple stances within the same pass, the maximum force values for that extremity were averaged. Impulse (Kg*sec) was the average of all foot strikes for the given extremity. Impulse (%BW*sec) was the average impulse of all foot strikes for a given extremity and normalized to body weight. Finally, maximum peak pressure (kPa) was defined as the peak force per unit area for a given extremity. To our knowledge, this device has not been validated for mixed breed goats with a significant range of weights; we present descriptive data for this population.

### Statistical analysis

Descriptive statistics for each parameter were generated, including the mean, standard deviation, and minimum and maximum values (IBM SPSS 25, Armonk, NY). Each gait parameter was analyzed with a Shapiro Wilk test to evaluate for normality of distribution (p-value > 0.05). An appropriate parametric student t-test or nonparametric Wilcoxon rank sum was performed for each variable between days to evaluate for repeatability between days. A one-way ANOVA followed by Tukey’s post-hoc test was performed to compare gait parameters between limbs. For variables that were not normally distributed, a one-way Kruskal-Wallis test followed by a pairwise comparison between limbs with a Dunn-Bonferroni correction to compare gait parameters was performed. A sample size estimate to detect the effect of time and extremity with 80% power was performed. For all statistical tests, a p-value of < 0.05 was considered significant.

## Results

Out of 46 goats, 33 (72%) met the inclusion criterion for analysis. Goats readily walked through the alleyway and across the pressure-sensing mat system without difficulty. Thirteen goats were removed from statistical analysis because of having incomplete data sets (lack of 2 valid passes within a maximum of 5 attempts). Due to the range in stance time (sec), swing time (sec), stride time (sec), and stride length (cm), it was necessary to average two valid passes to obtain consecutive footfalls for a complete data set for each test day. After the repeatability assessment, data was then pooled. Pooled data for stance-stride variables were tabulated (Tables 2 and 3). Descriptive statistics for gait variables are reported as the means ± standard deviation and range (minimum, maximum) for 33 goats having complete datasets. The mean number of stances was 9.03±1.81 (6.00, 14.00). The gait-time front (sec) was 1.73±0.72 (0.51, 4.10), the gait distance-front (cm) was 132.38±13.92 (96.90, 153.00), and the gait velocity-front (cm/sec) was 92.64±36.30 (34.40, 279.60). The mean gait cycle time (sec) for 33 goats was 1.00±0.25 (0.44, 2.00), while the mean cycles/minute was 64.67±15.62 (30.00, 139.00).

**Table 2:** Descriptive statistics for the stance gait parameters. Results are displayed as mean ± standard deviation (SD) followed by a range (minimum, maximum).

Extremity (N = 33)	Stance Time (sec)	Swing Time (sec)	Stride Time (sec)	Stride Length (cm)	Stride Velocity (cm/sec)
Limb	Mean ± SD (min., max.)	Mean ± SD (min.,max.)	Mean ± SD (min.,max.)	Mean ± SD (min., max.)	Mean ± SD (min., max.)
Left Front	0.68±0.23 (0.20, 1.23)	0.37±0.14 (0.17, 0.98)	1.00±0.26 (0.30, 2.05)	81.95±14.60 (45.50, 111.40)	90.13±40.63 (34.40, 334.30)
Left Hind	0.67±0.25 (0.28, 1.49)	0.36±0.17 (0.09, 1.19)	1.03±0.30 (0.43, 1.87)	77.23±20.51 (18.70, 122.40)	85.20±41.93 (15.70, 294.50)
Right Front	0.66±0.23 (0.25, 1.48)	0.37±0.12 (0.21, 0.85)	1.02±0.28 (0.48, 1.90)	83.82±14.41 (57.80, 119.00)	92.30±35.25 (35.20, 247.90)
Right Hind	0.68±0.27 (0.28, 1.81)	0.35±0.12 (0.13, 0.82)	1.00±0.24 (0.50, 1.61)	78.17±17.86 (32.90, 114.80)	84.96±33.17 (24.70, 218.90)

**Table 3:** Descriptive statistics for the stride gait parameters. Results are displayed as mean ± standard deviation (SD) followed by a range (minimum, maximum).

Extremity (N = 33)	Maximum Force (%BW)	Maximum Force (Kg)	Impulse (%BW x sec)	Impulse (kg x sec)	Maximum Peak Pressure (KPa)
Limb	Mean ± SD (min., max.)	Mean ± SD (min., max.)	Mean ± SD (min., max.)	Mean ± SD (min., max.)	Mean ± SD (min., max.)
Left Front	46.89±10.92 (24.80, 71.70)	24.38±5.22 (10.18, 35.86)	22.97±8.82 (9.20, 57.90)	12.23±5.09 (3.75, 28.94)	109.44±25.28 (42.00, 212.00)
Left Hind	34.61±7.58 (17.70, 50.50)	18.01±4.56 (7.26, 28.29)	16.30±7.02 (5.80, 39.00)	8.64±4.25 (2.39, 20.53)	92.55±20.49 (37.00, 144.00)
Right Front	47.21±9.10 (27.50, 65.10)	24.49±4.68 (11.88, 35.14)	22.89±8.14 (7.30, 47.70)	11.87±4.57 (3.66, 27.71)	107.97±23.05 (49.00, 176.00)
Right Hind	35.98±8.77 (20.50, 54.40)	18.89±4.76 (8.39, 34.77)	16.92±6.23 (6.6, 35.70)	9.07±3.84 (2.73, 18.57)	92.89±22.53 (39.00, 157.00)

Data that were normally distributed included maximum force (%BW), maximum force (Kg), stride length (cm), and maximum peak force (KPa; p > 0.05). Of those parameters, maximum force (%BW), maximum force (Kg), and maximum peak force (KPa) were significantly different among the limbs (p < 0.001).There was no significant difference in stride length (cm) among the limbs (p > 0.05). As the gait parameters maximum force (%BW), maximum force (Kg), and maximum peak force (KPa) may be most associated with future orthopedic or animal welfare studies, they were selected for the sample size estimate analysis to detect the effect of time and extremity. Based on these variables, only 12 animals were needed to detect the effect of time and extremity.

When evaluating day 1 versus day 3, there were no significant differences for the variables maximum force (%BW), maximum force (Kg), impulse (%BW *sec), impulse (Kg*sec), or maximum peak pressure (KPa; p > 0.05) for any of the extremities. There was a day-effect for the variables stance time, stride time, stride length, and stride velocity. For the variable stance time (sec), there was a significant decrease in time for the majority of goats (n = 21, left front; n = 23, right front; n = 23, right hind) on day 3 as compared to day 1 (p < 0.05). For the variable stride time (sec), there was a significant decrease in time for the majority of goats (n = 19) on day 3 as compared to day 1 for the right hind limb (p < 0.05). For the variable stride length (cm) there was a significant difference for the left front and right front means with the left front being greater on day 3 versus day 1 and the right front mean being greater on day 3 versus day 1 (Table 3; p < 0.05). For the variable stride velocity (cm/sec) there was a significant difference for all extremities on day 1 versus day 3, with the means for the left front being greater on day 3, the means for the left hind being greater on day 3, the right front means being greater on day 3, and the right hind means being greater on day 3 (p < 0.05; Table 4).

**Table 4:** Students t-test evaluating day 1 versus day 3 for the variables stride length (cm) and stride velocity (cm/sec). * indicates no significant difference between days.

Extremity (N = 33)	LF			RF			LH			RH		
Runs	Day 1	Day 3	Mean +/- SD	Day 1	Day 3	Mean +/- SD	Day 1	Day 3	Mean +/- SD	Day 1	Day 3	Mean +/- SD
Stride Length (cm)	78.07	85.83	7.76 +/-	78.82	88.81	9.99 +/-	75.97	78.48	2.51 +/-*	74.58	81.75	7.17 +/-*
Stride Velocity (cm/sec)	79.91	100.35	20.44+/-	81.34	103.25	21.91 +/-	75.79	94.62	18.83 +/-	74.72	95.21	20.48 +/-

The mean stride length (cm) was greater on day 3 as compared with day 1 for the left front limb and right front limb (p < 0.05). The left hind and right hind limb means are reported with * as they were not significantly different between days. The mean stride velocity (cm/sec) was greater on day 3 as compared to day 1 for all extremities (p < 0.05).

**Fig 2:**
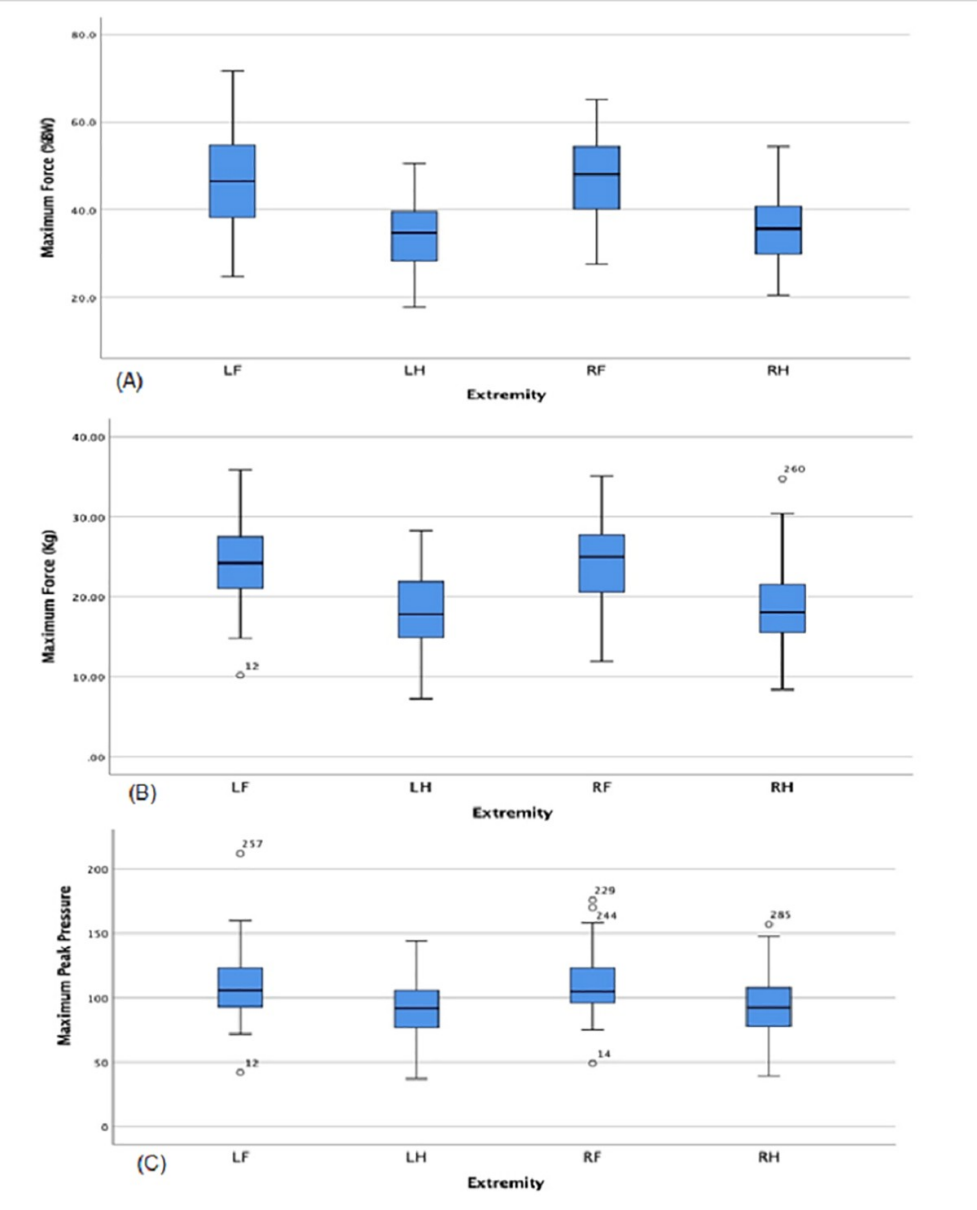
Box plots for Maximum Force (%BW), Maximum Force (Kg), and Maximum Peak Pressure (KPa). The line in the middle of each box represents the median value for each extremity for each variable. Upper and lower quartile ranges are also represented either above or below the line within the box itself as well as the entire range represented by the lines (whiskers). Outliers are represented as circles above or below the whiskers. (A) Maximum force (%BW) shows that the paired left front and right front means are greater than the left hind and right hind means (p < 0.001). (B) Maximum force (Kg) shows evidence that paired left front and right front means are again greater than paired left hind and right hind means(p < 0.001). Finally, in panel (C), Maximum Peak Pressure (kPa) is shown to be significantly different among the extremities with the paired forelimbs being greater than the paired hindlimbs (p < 0.001).

Within paired limbs, there were no significant differences between the means of the left front and right front limbs, nor the left hind and right hind limbs, respectively (p > 0.05). Significant differences were found for maximum force (Kg) between the limbs. There was a significant difference between the left front and hind limbs with the left front mean being greater than the left hind and right hind limb (p < 0.001). There was a significant difference between the right front limb and the hind limbs with the right front limb being greater than the left hind and right hind limb (p < 0.001; Fig 2B). Significant differences were found for maximum force (normalized to %BW; p < 0.001) between the limbs. There was a significant difference between the left front and left hind and the hind limbs with the left front mean being greater than the left hind and right hind limb (p < 0.001). There was a significant difference between the right front and the hind limbs’ weight, the right front mean being greater than the left hind and right hind limb (p < 0.001; Fig 2A). A significant difference was found between the limbs for maximum peak pressure (kPa). The left front limb was significantly greater than the left hind and right hind limb (p<0.001). The right front limb was greater than the left hind and right hind limb (p < 0.001; Fig 2C).

**Fig 3:**
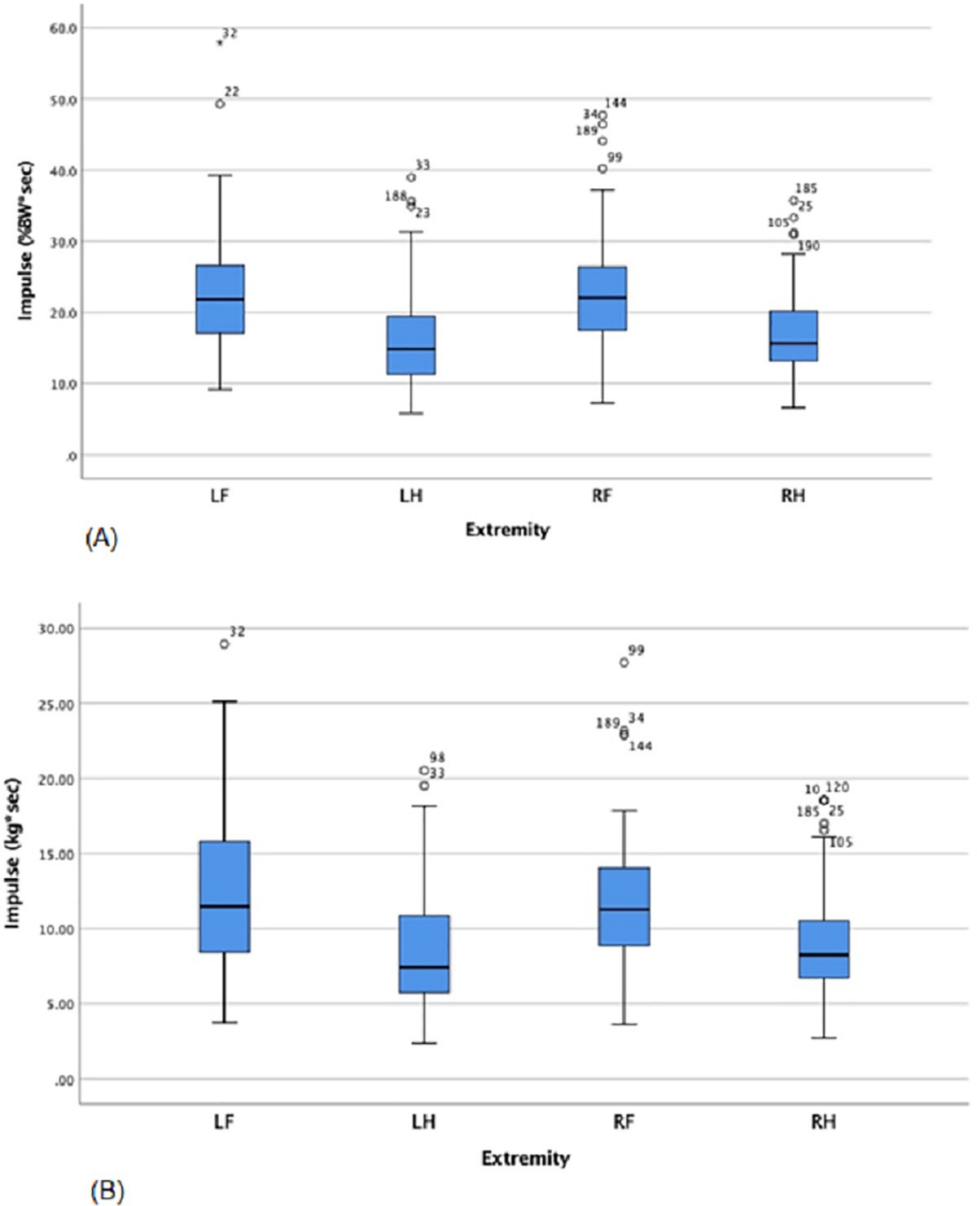
Box plots for Impulse (%BW*sec) and Impulse (Kg*sec). The line represents the median value for each extremity for each variable. Upper and lower quartile ranges are shown and the entire range is represented by lines (whiskers) with outliers being represented as circles either above or below the whiskers. (A) Impulse (%BW*sec) is significantly greater for the paired forelimbs than the paired hind limbs (p < 0.001). (B) Impulse (kg*sec) is significantly greater for the paired forelimbs than the paired hind limbs (p < 0.001).

No significant differences were found between the extremities for the gait parameters stance time (sec), swing time (sec), stride time (sec), and stride velocity (cm/sec; p > 0.05). Significant differences were found among between the extremities for the gait parameters impulse (%BW*sec) and impulse (kg*sec; p < 0.001). Impulse was significantly less for the paired hind limbs compared with the paired forelimbs (p < 0.001; Fig 3B). The impulse normalized to percent body weight was significantly less for the paired hind limbs compared with the paired forelimbs (p < 0.001; Fig 3A).

## Discussion

In the present study, we were able to quantitatively assess biometric variables of gait and associated forces during ambulation in goats free of lameness. We found that goats could be quickly trained to walk across a pressure-sensing walkway, allowing consistent recordings of data for variables of stride, gait, and force. Based on the results of this study, we found that data derived for stride variables is more susceptible to variations when assessments are done at multiple time points. Also, front limb and rear limb assessments should be evaluated separately when using force variables to serially evaluate goat ambulation. We were able to describe the characteristics associated with stride and weight-bearing forces in goats free of lameness. Thus, the consequences of this study are important in that they provide a baseline for future studies using a pressure-sensing mat investigating lameness.

Detailed knowledge of gait characteristics in goats may benefit the veterinary community in that lameness evaluation of small ruminants may now be more easily done at an objective level, allowing this technology to potentially be used in herd health situations. Our results suggest that biometric pressure sensing may be a useful tool for gait assessment in goats regardless of breed. Using a pressure sensing system, we were able to quantitatively assess gait and biometric forces during ambulation in goats free of lameness using a pressure-sensing mat as a biometric tool for gait analysis. It is important to note that baseline clinical health examinations were not included as confounding variables in this study. Due to our interests in orthopedic research, which typically do not include infirmed, immature, or geriatric animals, we limited our population to young, skeletally mature goats free of lameness [1]. It was assumed that this population included mainly healthy goats. Currently, we do not have baseline values for goats that are clinically evaluated to be ill or outside the skeletal range we have evaluated. Future studies would need to include a correlation between visual lameness scores and alterations from the baseline values reported here

Pressure sensing systems evaluate ground reaction forces where pressure causes activation of the sensors [34]. Quantitative measurements may be useful to determine functional weight bearing and to assess changes in acute weight bearing or for serial evaluation of pain [35] The number of walking passes needed to obtain valid data sets may vary; we were able to obtain data using the pressure sensing walkway set-up within an alleyway with relatively few repeated walking trials [36]. We were successful in being able to collect complete data sets with relatively few passes in the majority of goats tested. The pressure-sensing mat was useful to measure functional gait characteristics even in the face of a nonhomogeneous population. With minor variations, the biometric data acquired was useful for statistical analysis within walking trials and between trial days allowing for pooled analysis. The walkway system provided a means of collecting valuable data with limited training while using a more realistic animal model population. Additionally, when weight bearing is used as an outcome variable, this accurate quantitative tool may help in power calculations of treatment group size when designing animal experiments.

Some caution may be warranted when evaluating the parameters stance time (sec), stride time (sec), stride length (cm), and stride velocity (cm/sec) between days, as these variables were not always repeatable between days among the extremities. This is particularly true for stride velocity (cm/sec), which was not repeatable for any limb between days. A solution to this in the future would be to standardize for stride velocity (cm/sec) as has been reported for other walkway assessment tools [31]. As we used a nonhomogeneous population, this standard was not met. A previously published study in which dogs of various sizes were allowed to walk with their preferred velocity, reliable gait assessment was obtained, indicating that perhaps standard velocity may not be necessary when assessing gait parameters for lameness [33]. Despite this limitation, we were able to obtain valid results that should be acceptable as quantitative data for gait analysis even when performed serially over time. One potential confounding variable is that the investigator was placed on the right side of the mat. Goats tended to walk closer to the left side of the mat, opposite from the data acquisition station. Similar to sheep, goats are prey species animals and tend to shy away from human contact [5, 11, 36]. This contributed to 13 goats being eliminated from the data set, as their behavior was unacceptable in that they were unable to walk forward without distraction or hesitation. This is consistent with dogs being walked on a leash and leash side influencing gait symmetry [33]. In these goats, multiple passes were discounted because of walking more quickly than desired or stopping halfway across the pressure mat. Additional training, such as walking in either direction, acclimatization, or a different alleyway system for acquiring ambulatory data, may help normalize this data. Despite goats being removed from study, significant results were able to be retained, similar to a pressure sensing study with turkeys [27]. While not all birds were available at all time points, significant results were able to be obtained indicating that data may be pooled for herd populations and gait parameters may be evaluated over time [27]. Finally, goats have relatively small hooves; therefore, using a pressure mat with a greater sensor density may improve precision and accuracy of weight bearing data.

Similar to other studies, we found that maximum force (Kg), maximum force normalized to body weight (%BW), and maximum peak pressure (kPa) was greater in the forelimbs than the hind limbs when measured using a pressure-sensitive walkway [31]. This is valuable for orthopedic research when using small ruminant models to establish methods for objective, quantitative assessment of weight bearing and gait [17, 31]. Providing a tool to supplement subjective VLS evaluations with objective data will allow more robust monitoring and assessment of gait in research subjects. The importance of providing an objective standard for lameness assessment in orthopedic models cannot be understated. Measuring weight bearing in fracture models is an expected standard of care; however, there remains a deficiency of literature on the topic [37]. Current animal use protocols, in general, use subjective measurements because of ease of application and lack of access to objective alternatives [38–41]. Moving towards nonbiased objective data is expected to benefit animal welfare and improve the quality of quantitative data in the use of small ruminants as models for orthopedic disease.

No validated visual or objective lameness assessment system exists for goats. Categorical assessment tools are subject to bias interpretation and must be analyzed using statistical tests for categorical data, making them less sensitive at identifying differences [5–12]. Difficulties with subjective data include inter-observer differences, limitations of categorical data, and lack of a standard scoring system for the species. Inter-observer differences have long been noted in lameness research in horses [41]. Objective assessment tools offer the possibility of validating subjective tools for use in research, which is currently limiting [41]. Analysis of categorical data, such as VLS scores, are challenging even after transformation to proportional (categorical) data and can yield spurious results [41]. Categorical data, therefore, requires different methods for analysis that may be less sensitive than objective, continuous data obtained with tools, such as pressure mat sensing systems [42]. Variability in subjective scoring systems, comparing lameness scores across any type of research model of lameness, becomes inherently problematic at a time where there is a call for large animal models to become more standardized [43, 44]. Other alternatives include the use of accelerometers to measure activity and define 3D gait characteristics, such as height of excursion of the limb/foot. These technologies are less adaptable to real-time analysis and assessment [37].

In the present study, we quantitatively assessed gait and biometric forces during ambulation in goats subjectively free of lameness using a pressure-sensing mat as a biometric tool for gait analysis. The pressure-sensing mat is an objective tool, free of inter-observer differences, and easy-to use. In an era where scientific procedures involving the use of animals is at an all-time low, the importance of providing stress free, accurate results when doing potentially painful procedures cannot be overstated [45].
